# Primordial germ cell-mediated transgenesis and genome editing in birds

**DOI:** 10.1186/s40104-018-0234-4

**Published:** 2018-01-31

**Authors:** Jae Yong Han, Young Hyun Park

**Affiliations:** 1 Department of Agricultural Biotechnology and Research Institute of Agriculture and Life Sciences, College of Agriculture and Life Sciences, Seoul National University, Seoul, 08826 South Korea; 2 Institute for Biomedical Sciences, Shinshu University, Minamiminowa, Nagano, 399-4598 Japan

**Keywords:** Avian, Genome editing, Primordial germ cell, Transgenesis

## Abstract

Transgenesis and genome editing in birds are based on a unique germline transmission system using primordial germ cells (PGCs), which is quite different from the mammalian transgenic and genome editing system. PGCs are progenitor cells of gametes that can deliver genetic information to the next generation. Since avian PGCs were first discovered in nineteenth century, there have been numerous efforts to reveal their origin, specification, and unique migration pattern, and to improve germline transmission efficiency. Recent advances in the isolation and in vitro culture of avian PGCs with genetic manipulation and genome editing tools enable the development of valuable avian models that were unavailable before. However, many challenges remain in the production of transgenic and genome-edited birds, including the precise control of germline transmission, introduction of exogenous genes, and genome editing in PGCs. Therefore, establishing reliable germline-competent PGCs and applying precise genome editing systems are critical current issues in the production of avian models. Here, we introduce a historical overview of avian PGCs and their application, including improved techniques and methodologies in the production of transgenic and genome-edited birds, and we discuss the future potential applications of transgenic and genome-edited birds to provide opportunities and benefits for humans.

## Background

**Fig. 1:**
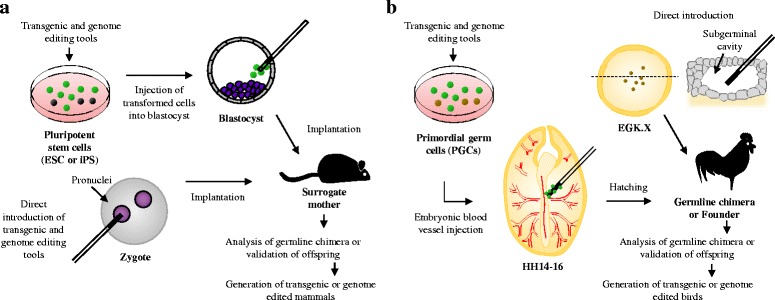
Transgenic and genome editing system in mammals and birds. **a** In mammals, transgenic (TG) and genome edited (GE) offspring can be produced via direct introduction of genome editing tool into the zygote or microinjection of genome edited ESCs into the recipient blastocyst. **b** In birds, TG and GE offspring can be produced via injection of genome edited PGCs into the blood vessel of recipient

The advancement of genetic modification tools and precise genome editing technologies has created a new era in which the genotype, phenotype, and traits of animals can be easily modified. Traditionally, animal breeders used selective breeding or artificial breeding strategies to improve productivity, food quality, and other traits of offspring through the selective mating of highly qualified parents [1]. In terms of the genomic DNA sequence of the desired animal, this selective breeding strategy is in line with effect of current genetic modification or genome editing. Thus, it has become possible to more efficiently improve and precisely manipulate the genetic traits of animal via recent genetic modulation technologies in combined with conventional breeding strategy. Currently, the introduction of genome modulation technology to a targeted animal inevitably requires germline modification of that animal, enabling the transmission of modified genetic traits to subsequent generations [2]. Germline modification strategies differ among animal species. In mammalian species, the first transgenic mouse was produced by microinjection of foreign DNA into the pronucleus of a fertilized oocyte [3]. The first genetically modified livestock, including rabbits, sheep, and pigs, were successfully produced in the same manner [4]. Even though the efficiency of developing founder animals is quite low and foreign DNA is randomly integrated into recipient genomes, this strategy is still a major technological method used in animal transgenesis. Another major method in mammalian transgenesis, especially in mice, is the use of germline competent cells like embryonic stem cells (ESCs) for germline modification (Fig. 1a). In mammals, germline chimeras that have a mixture of germ cells originated from both endogenous and exogenous germ cells can be produced via injection of genetically modified ESCs into recipient blastocyst [5, 6]. Through the testcross analysis of germline chimera, genetically modified ESC-mediated transgenic offspring can be generated. However, unlike mammals, birds have a unique transgenesis and genetic modification system (Fig. 1b) due to their oviparity and the physiological properties of the ovum [7]. Since avian zygote shows discoidal meroblastic cleavage with a large amount of yolk and a small germinal disc, it is difficult to introduce foreign DNA into zygote and microinject avian ESCs into blastoderm [8–10]. The first transgenic avian specimen was a chicken that was produced via sub-germinal cavity injection of a retroviral vector into an Eyal-Giladi and Kochav (EGK) [11] stage X embryo [12]. Since then, various strategies have been suggested for producing genetically modified transgenic birds, including viral infection into stage X embryos [13–15], microinjection of transgenes into fertilized eggs [10, 15], and embryonic stem cells [16]. However, due to low germline transmission efficiency, these methods are not successful in producing genome-modified birds via homologous recombination until recently. To overcome this limitation, much effort has focused on the utilization of primordial germ cells (PGCs) as an alternative strategy comparable to mammalian germline-competent ESCs [17]. Here we present an overview of PGCs and recent progress in transgenesis and genome editing technology, and introduce potential strategies for PGC-mediated genetic modulation in birds.

### Historical overview of avian primordial germ cells

#### Origin, specification, and development of primordial germ cells

**Fig. 2:**
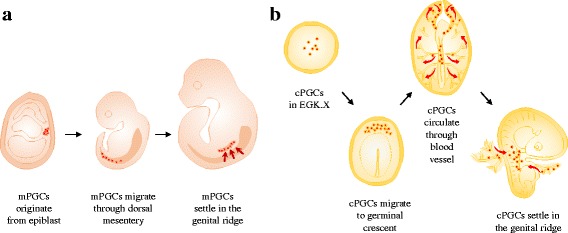
Schematic representation of the development and migration of PGCs in mouse and chicken. **a** Mouse PGCs originated from epiblast, and migrate through dorsal mesentery to seettle in the genital ridge. **b** Chicken PGCs located at the center of area pellucida region, and they migrate through germinal crescent and vascular system to settle in the genital ridge

In late nineteenth century, Waldeyer first observed the origin of germ cells in the germinal epithelium of chicken embryos [18]. Thereafter, Swift reported that avian PGCs arose from the endodermal region, the so-called germ wall [19]. Avian PGCs are observed in the epiblast layer and hypoblast in the central region of the area pellucida of EGK stage X blastoderm [11, 20, 21]. During early embryogenesis in chicken (Fig. 2a), PGCs migrate from the central region of the area pellucida toward the germinal crescent region until Hamburger and Hamilton (HH) stage 4 [22–24]. After formation of the primitive streak, PGCs are observed in the germinal crescent region of an extraembryonic site at HH stages 4–10 [11, 23, 25]. Subsequently, PGCs located at the anterior region enter the vascular system of extraembryonic blood vessels via the anterior vitelline vein during HH stages 10–12 [26, 27], and they start to settle in the gonadal anlagen at 2.5 d of incubation [28]. On the other hand, mouse PGCs originate from proximal epiblast and specified via bone morphogenetic proteins (BMP) signaling derived from the extraembryonic ectoderm and visceral endoderm [29]. During mouse embryogenesis (Fig. 2b), PGCs move from posterior primitive streak to endoderm, and subsequently migrate from hindgut endoderm to the mesentery, and finally settle in the genital ridge [30, 31]. When compared to mouse PGCs, the unique migratory pathway of avian PGCs enables us to develop PGC-mediated germline transmission and transgenic system in birds.

PGCs have a large amount of cytoplasmic glycogen granules. Therefore, periodic acid-Schiff (PAS) staining is conventionally used to identify PGCs in chick embryos [32], and Eyal-Giladi et al. suggested that PGCs originated from the epiblast around EGK stage X based on PAS staining results [33]. Because there were no specific molecular markers of PGCs or germ plasm, avian species had been assumed to follow the induction mode of PGC specification [34–36]. However, after the discovery of the chicken vasa homolog (*CVH*) gene and the tracing of its expression pattern from the oocyte through all developmental stages, it was revealed that avian germline specification is determined by maternally inherited factors, which strongly suggests that avian PGCs follow the germ plasm model of specification [37]. Moreover, a recent study on tracing chicken deleted in azoospermia-like (*DAZL*) gene in intrauterine-stage chicken embryos reinforces the evidence for a germ plasm model of avian PGC origin and specification [38].

#### Isolation and culture of primordial germ cells

Avian PGCs can usually be isolated at three different developmental stages, including in the germinal crescent of HH stage 4–8 embryos, vascular system of HH stage 14–16 embryos, and gonadal ridge of HH 26–28 embryos. Before the discovery of PGC cell-surface markers, PGCs were isolated using a density gradient-dependent centrifugation method [39, 40]. However, the utility of this method for isolating PGCs was limited due to low yield rates, purity, and viability after isolation. After the identification of PGC-specific surface antigens such as stage-specific embryonic antigen-1 (SSEA1) in chickens and quail germ cell-specific marker (QCR1) in quail, it is possible to collect highly purified avian PGCs using magnetic-activated cell sorting (MACS) or fluorescence-activated cell sorting (FACS) systems via PGC-specific antibodies [41–43]. However, it is still difficult to isolate the PGCs of wild or endangered birds using such cell sorting methods, as their PGC-specific surface markers have not yet been identified. Accordingly, Jung et al. recently developed a transwell-mediated size-dependent isolation method for various avian PGCs in HH stage 14–16 embryonic blood, a strategy based on the size of PGCs [44].

Since the in vitro long-term culture of PGCs was successfully established by van der Lavoir in 2006 [45], much effort has been focused on optimizing PGC culture systems and cell signaling mechanisms for the in vitro proliferation of PGCs while maintaining their germline competency. It was subsequently revealed that basic fibroblast growth factor is an essential factor for in vitro proliferation and survival via the MEK/ERK cell signaling pathway [46, 47]. Recently, Whyte et al. [48] demonstrated that the in vitro self-renewal of PGCs requires MEK1, AKT, and SMAD3 cell signaling to maintain germline competency, and Lee et al. [49] found that Wnt/β-catenin signaling is also required for the proliferation of PGCs in vitro. In the near future, PGC culture systems should be developed for multiple bird species and optimized for the application of PGC-mediated avian transgenesis and genome editing.

#### Production of germline chimeras via primordial germ cells for avian transgenesis

**Fig. 3:**
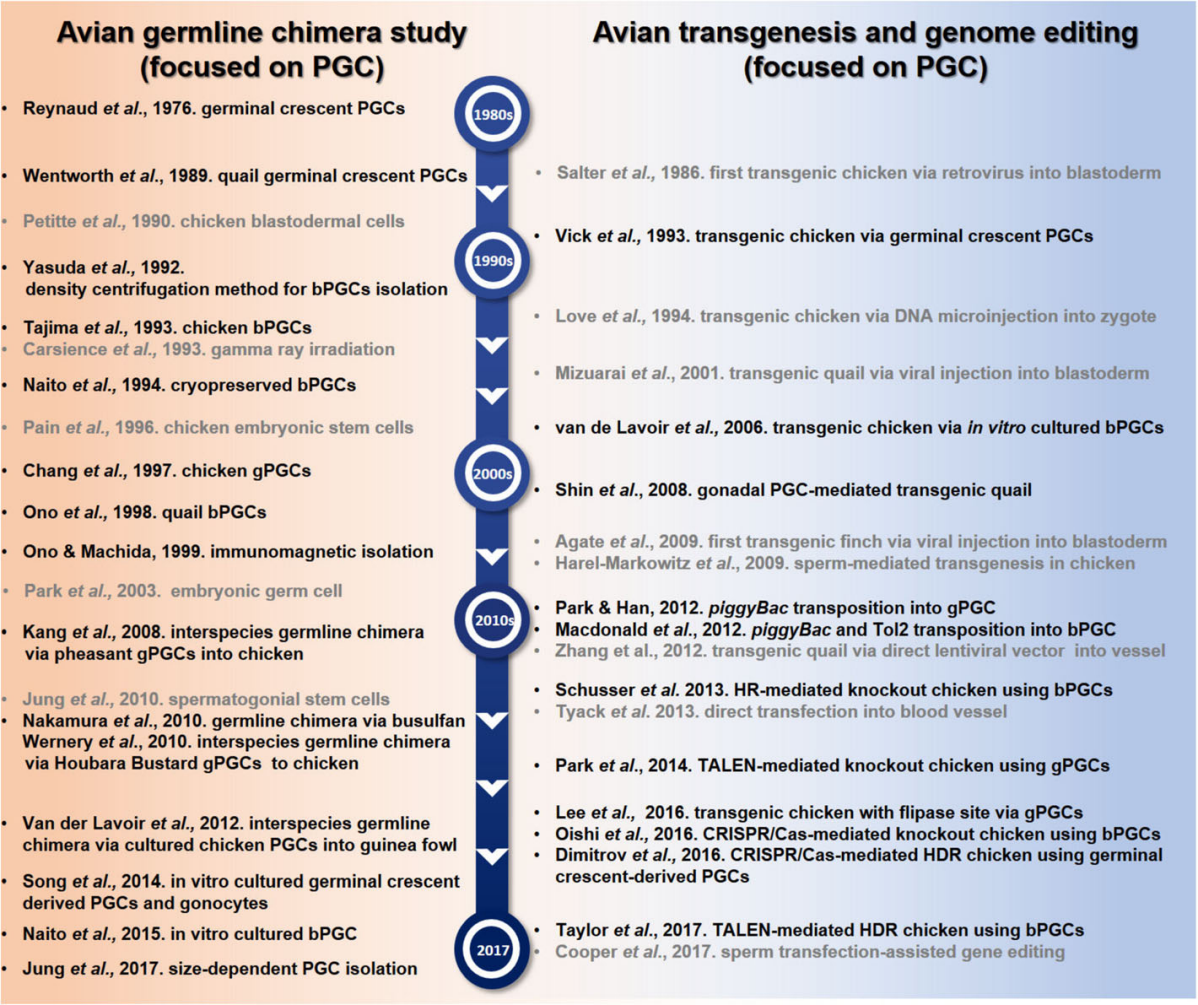
Historical contributions to advancemnet of primordial germ cell-mediated production of germline chimeras and genetic modulation in birds. PGC, primordial germ cell; bPGC, embryonic blood-derived PGC; gPGC, embryonic gonad-derived PGC; HR, homologous recombination; TALEN, transcription activator-like effector nuclease; CRISPR/Cas9, clustered regularly interspaced short palindromic repeat (CRISPR)-CRISPR associated protein; HDR, homology-directed repair

“Germline chimera” usually refers to the presence of mixed gametes from different breeds or species in one individual. For the production of highly efficient transgenic birds, much effort has been focused on improving the efficiency of germline transmission. In 1976, Reynaud observed the colonization of germinal crescent-derived donor turkey PGCs in recipient chicken gonads after intravascular injection and produced a germline chimera chicken that produced functional gametes derived from turkey primordial germ cells [50]. PGCs isolated from quail germinal crescent were later successfully transferred to recipient embryos to produce quail germline chimeras [51]. Subsequently, the first transgenic bird was produced using PGCs isolated from the germinal crescent of HH stage 5 chicken embryos [52]. As shown in Fig. 3, avian germline chimeras and donor-derived progeny have been produced by transferring PGCs isolated from the blood of HH stage 14–16 embryos (bPGCs) [53, 54] and gonads of HH stage 26–28 embryos (gPGCs) [55, 56] in chicken and quail. As previously mentioned, density gradient centrifugation and immunomagnetic cell sorting methods were developed to obtain purified PGCs and efficiently produce germline chimeras [39, 42]. In the meantime, germline chimeras were produced using cryopreserved bPGCs [57] and gPGCs [58]. Cryopreservation of PGCs can enable the preservation of avian genetic resources and restore endangered bird species. Recently, interspecies germline chimera have been produced for the restoration and preservation of birds via transplantation of pheasant PGCs [59] and Houbara Bustard PGCs [60] into chicken or chicken PGCs into guinea fowl. Meanwhile, there are other efforts to produced germline chimera more efficiently through depletion of endogeneous PGCs of recipient embryo. Various methods have been used to eliminate the endogeneous germ cells in birds through exposure to gamma ray [61], administration of busulfan into embryo [62] and removal of blood from recipient embryos at HH stages 14–15 [57]. In 2010, Nakamura et al., reported that the germline chimera efficiency of busulfan-treated founder was about 99%, whereas the efficiency of busulfan-untreated chimera was about 6% [63]. Thus, strategies for depletion of enodogenous PGCs can promote the development of transgenic and genome edited birds efficiently. On the other hand, there have been many effort to develop alternative germline chimera systems without PGCs, using other germline competent cells including blastodermal cells [64], embryonic germ cells [65], germline stem cells, and spermatogonial stem cells [66]. However, their germline transmission efficiency is quite low compared to PGC-mediated germline chimera system. Because germline chimeras and genetically modified chickens can be produced using in vitro cultured PGCs in chickens [45], the in vitro culture system of PGCs has been optimized and the germline competency of in vitro cultured PGCs has subsequently been revealed [46, 47, 67]. Although the germline transmission efficiency was quite variable, from 0% to about 100% for each PGC line, PGCs are still regarded as the most optimal germline-competent cells that can be expanded in vitro without loss of germline competency. To produce more efficiently germline chimeras using PGCs, several effort have been made to enhance the germline competency of PGCs via optimization of culture condition of PGCs [48, 49, 67–69]. However, the relationship between in vitro culture of PGC and loss of germline competency is still unclear, and the systems related in vitro long-term culture of competent PGC is inadequate at present. In addition, it may be required to identify best germline competency-associated marker, which contribute to enhance the quality of PGCs. Although there are still challenges to overcome, the PGC-mediated germline transmission system is the most efficient way to produce transgenic and genome-edited birds at present.

### Genetic modification and genome editing in birds

#### Overview of transgenesis in birds

Prior to the establishment of long-term in vitro PGC culture systems, the major transgenic technology used in birds was based on injecting viruses into EGK stage X embryos. In avian species, the first transgenic chicken was produced by microinjection of recombinant avian leukosis viruses into the subgerminal cavity of EGK stage X embryos [12]. Subsequently, Vick et al., successfully produced transgenic chicken using genetically modified PGCs via retrovirus [52] In addition, Mizuarai et al., produced transgenic quail using direct injection of a replication-defective retroviral vector into the blastodermal stage embryos [70]. Because randomly integrated transgene in genome of transgenic animal was frequently silenced [13, 70–72], the lentiviral system was introduced to avian transgenesis as an efficient viral transduction system. It successfully produced various transgenic chickens without any gene silencing [73–76]. Furthermore, Agate et al., produced first green fluorescent protein (GFP)-expressing transgenic finch using microinjection of lentivirus into blastodermal stage embryos [77]. Meanwhile, Shin et al., successfully produced transgenic quails using gPGCs-mediated germline transmission via lentiviral system [78]. Although the efficiency of gPGC-mediated transgenesis was similar to blastoderm-mediated transgenesis in quail, it has been enabled to produce transgenic birds via viral transfection combined with directly purified PGCs without cultivation.

On the other hand, there have been many efforts to develop non-viral transgenic systems without PGCs, such as sperm-mediated gene transfection [79, 80] and direct microinjection of transgenes into the fertilized eggs [81]. However, these strategies showed low germline transmission efficiency compared to PGC-mediated transgenesis. Due to the establishment of long-term in vitro culture systems, PGC-mediated transgenesis has become a more optimal method for developing genetically modified birds than the aforementioned methods. Accordingly, a highly efficient non-viral system for stable genomic integration of transgenes into the genome of PGCs was developed using transposable elements, such as piggyBac and Tol2 [82, 83]. The introduction of transgenes into the genomes of cultured PGCs using lipofectin or electroporation showed a remarkably higher efficiency than the conventional methods for producing transgenic chickens. More recently, a piggyBac transposon system with Flipase recombinase recognition sequences was developed for introducing site-specific gene cassette exchange in transgenic chicken genomes via PGCs [84]. Meanwhile, there have been several efforts to develop alternative strategies for transgenesis without the use of PGCs. Although the level of transgenic efficiency is usually lower than PGC-mediated transgenesis, the transgenic birds were produced via direct injection of transfection reagents into circulating PGCs at HH stages 14–16 [85–87]. This strategy can be applied to produce genetically modified birds, of which PGCs are difficult to manipulate in vitro.

#### Precise genome editing technology

In recent years, investigators have successfully developed efficient systems for precise genome editing using programmable nucleases, including zinc-finger nucleases (ZFNs), transcription activator-like effector nucleases (TALENs), and clustered regularly interspaced short palindromic repeat (CRISPR)-CRISPR associated protein (CRISPR/Cas). Compared to conventional genetic modification technology based on homologous recombination events, which have extremely low frequency in eukaryotic cells [88], these programmable nucleases yield a much higher frequency of homologous recombination events [89] and also induce targeted mutagenesis through error-prone non-homologous end-joining (NHEJ) [90]. Because these programmable nucleases share common features with conventional genetic engineering tools, including DNA double-strand break repair, gene disruption, gene insertion, gene correction, and point mutagenesis [91], programmable nucleases are innovative genome editing tools. ZFNs were first discovered in 1996 and consist of a zinc finger-based DNA binding domain for DNA recognition and a *Fok*I nuclease for DNA cleavage [92]. ZFNs have been used in several organisms for gene editing, including mouse, rat, and zebrafish [93, 94], but there are no reports of generating ZFN-mediated gene-edited birds. As a second generation programmable nuclease system, TALENs have a similar protein structure to ZFNs, consisting of a *Fok*I endonuclease and a DNA-binding domain, but they have different DNA-binding domains known as transcription activator-like effectors (TALEs), which can be programmed to bind targeted DNA sequences [95]. Although TALEN-targeted DNA sequences must start with a thymine base [96], the TALEN system is much more convenient for determining target sites than ZFNs. Accordingly, TALENs have been more widely utilized in various species due to easy construction, widely applicable possibilities [97, 98], and lower cytotoxicity than ZFNs [99]. A third generation programmable nuclease system is based on a CRISPR with a Cas endonuclease derived from the RNA-based immune system of prokaryotes against bacteriophages, viruses, or foreign nucleic acids [100]. In 2012, Jinek et al. reported that a dual RNA, called a guide RNA (gRNA), consisting of a 20-bp CRISPR RNA (crRNA) and universal *trans*-activating crRNA (tracrRNA), together with *Streptococcus pyogenes* type II Cas9 protein (Cas9), induced cleavage of specific target DNA sequences [101]. Thus, Cas9 coupled with dual RNAs has become a powerful tool for gene editing due to its target-specific cleavage capacity. In the CRISPR/Cas system, the target site selection depends on the protospacer adjacent motif (PAM) sequence NGG, which has an important role in the initiation of Cas9 nuclease activity [102, 103]. Compared to TALEN, CRISPR/Cas9 is simpler, easier to use for constructing chimeric single-guide RNA [104], and has lower cytotoxicity and higher targeting efficiency [105]. To enhance target specificity, avoid breakage of double-stranded DNA, reduce off-target effects, and increase homology directed repair (HDR) events or base conversion, various Cas9 variants such as Cas9n [106], Cas9dn [85], and Cas9 D10A [107] have been developed. In addition to the Cas9 endonuclease, a class 2-type V CRIPSR effector endonuclease called CRISPR from Prevotella and Francisella 1(Cpf1) was recently identified [108] which lacks tracrRNA and utilizes a thymidine-rich PAM recognition sequence, in contrast to the guanine-rich PAM sequence of the class 2-type II effector nuclease Cas9. Although it is difficult to directly compare the effectiveness of Cpf1 and Cas9 because of their different PAM sequences, genome-wide analysis shows that Cpf1 has higher accuracy and specificity and has relatively fewer off-target effects than Cas9 [109, 110]. Researchers should choose and use programmable nucleases appropriately for their own purposes, optimizing for factors such as no dsDNA breaks, higher HDR, lower off-target effects, or precise base conversion.

#### Generation of genome-edited birds: analysis from the germline transmission perspective

Despite the importance of avian species as an ideal animal model of early embryogenesis and organogenesis in developmental biology [111], it had been difficult to investigate loss or gain of function in specific genes in birds due to the lack of precise gene targeting system. Unlike mammalian species, specific gene-targeted birds could not be successfully produced until an in vitro culture system for PGCs and efficient gene editing technologies were developed (Fig. 3). In 2013, the immunoglobulin gene knockout chicken was first produced via homologous recombination in chicken PGCs [112]. The total germline transmission rate of targeted PGCs is approximately 0.1% because the homologous recombination event occurs at a very low frequency, as previously discussed. However, with recent advances in gene editing technology using programmable nucleases, the ovalbumin gene-targeted chicken was generated with TALEN in 2014 [113]. Although 8% of the chicks of the donor PGC-derived offspring were mutants from the transplantation of an average of 33.3% mutant PGCs, TALEN-mediated gene knockout showed higher germline transmission efficiency in mutant progeny than the conventional homologous recombination-mediated gene knockout system. This is because TALEN-induced NHEJ occurs much more frequently than homologous recombination in eukaryotic cells [91]. Subsequently, the CRISPR/Cas9 system-mediated ovomucoid (*OVM*) gene-targeted chicken was efficiently produced by transplanting transient puromycin-selected PGCs into endogenous PGC-ablated recipient embryos with gamma-ray irradiation [114]. In that report, the two G0 founders, with the exception of one founder, had on average 93% mutant semen, indicating that the CRISPR/Cas9 system induced *OVM* mutation was highly efficient in almost all of the donor PGCs. Furthermore, from the testcross analysis of two G0 founders, the donor PGC-derived offspring were 72%, of which 53% were *OVM* gene mutant offspring. Concurrently, Dimitrov et al. successfully produced CRISPR/Cas9-mediated precise genome-edited chickens via HDR insertion of an additional loxP site into the variable region segment segment (*VH*) of a loxP previously inserted into the joining gene segment (*JH*) of chicken immunoglobulin heavy chain (*IgH*) locus [112, 115]. Through Cre recombination of the loxP site inserted at the *IgH* locus, an approximately 28-kb genomic DNA sequence at the *IgH* locus was deleted. From their results, germline transmission rates were highly variable for each PGC line; even a founder from the same PGC line showed 0–90% efficiency. It is therefore important to use reliable germline-competent PGC lines for germline transmission of genetically modified or precisely edited genes. More recently, Tayler et al. successfully produced a *CVH* gene-targeted chicken via the TALEN-mediated HDR system, which induced GFP transgene integration into the *CVH* locus on the Z chromosome [116]. The efficiency of HDR-mediated GFP transgene knock-in in the *CVH* locus was 8.1% in two-week recovered PGCs after two days of puromycin selection. Although the percentage of GFP-integrated PGCs used to generate the G0 founder was not reported, they established stable GFP-knock-in PGCs using puromycin selection for two weeks. They produced 6% *CVH*-targeted offspring from one G0 male founder that had 10% genomic equivalents in its semen. From the TALEN and CRISPR-mediated genome editing results, the germline transmission efficiency of G0 founders vary among each genome edited PGC lines. In this regards, it is also important to optimize the conditions for stable PGC lines while maintaining their germline competency even after genetic modification and gene editing, because PGC lines seem to have different germline competencies for each established cell line and lose their germline competency during long-term in vitro cultivation and genetic modification [67, 68, 117].

Meanwhile, Cooper et al. reported an ideal method for avian genome editing called sperm transfection-assisted gene editing, which is based on direct delivery of a CRISPR gRNA and Cas9 mRNA mixture into spermatozoa [118]. This method shows a targeting efficiency from 0 to 26.6% mutation in the GFP gene and from 0 to 3% mutation in the doublesex and mab-3 related transcription factor 1 (*DMRT1*) gene. Although the efficiency of gene editing and germline transmission is still low compared to other current PGC-mediated transgenesis and genome editing methods, this strategy can be utilized as a potential alternative for avian transgenesis and genome editing without culturing PGCs in birds, of which PGCs is difficult to manipulate in vitro.

### Application of genome editing technology in birds

**Fig. 4:**
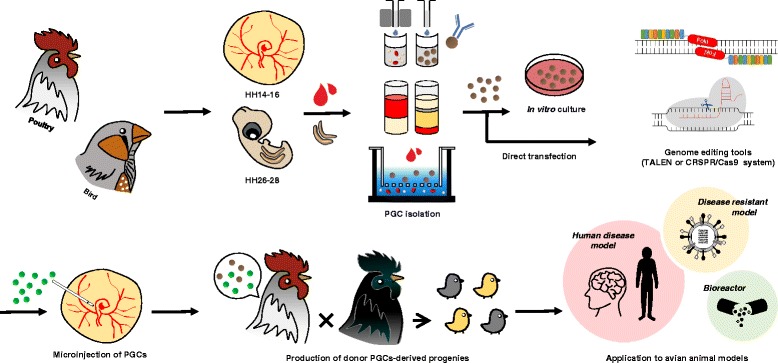
Strategies for the production of genome-edited birds. Avian PGCs can be isolated from embryonic blood (HH stages 14–16) and embryonic gonads (HH stage 26–28) by cell-surface antibody-mediated methods, density gradient centrifugation, and size-dependent isolation methods. Genome-edited birds can be produced by transplanting directly isolated or in vitro cultured PGCs into the blood vessels of recipient embryos after the introduction of genome editing tools. Avian genome editing systems can be applied to produce various avian models, such as avian disease resistance models, bioreactor models, and human disease models

The chicken genome sequencing project was completed in 2004, and chicken genomic sequences have been available to the public since that time [119]. Subsequently, the genomic sequences of the zebra finch and turkey have also been made accessible. [120, 121]. Due to recent next generation sequencing technologies, the bird 10K genome sequencing project has been initiated in 2015. Furthermore, the Earth BioGenome Project has recently been proposed to sequence the DNA of all life on Earth, which will covers the genomic information of 1.5 million species [122]. As the genomic information of various avian species has been revealed, it will create infinite possibilities and provide multiple opportunities to access invaluable genetic information from birds [123]. Until recently, there was no way to utilize this valuable avian genetic information in developing genome-edited birds, because there was no efficient genome editing system that could be practically used in birds. The recent progress in genome editing technology in birds via PGCs has ushered in an innovative era of avian genome manipulation for the development of invaluable avian models (Fig. 4). First of all, in chickens, we expect to be able to create an efficient bioreactor system for producing valuable proteins by applying gene editing technology. It is well known that as potential bioreactors chickens have the key benefits that egg white protein is easy to purify and they produce a large amount of egg white protein daily [7, 124]. Although the strategy for developing chickens as bioreactors has focused on the production of target proteins using the ovalbumin promoter, which is the most powerful promoter of egg white proteins [76, 125], it is possible to directly integrate a target protein sequence into the ovalbumin locus via HDR-mediated gene editing. This HDR-mediated target protein insertion into the ovalbumin locus could ultimately be an ideal bioreactor system producing more than one grams of target protein from a single egg with low cost. Genome editing in chickens is also expected to remove or enhance specific nutrients in the meat and eggs of chickens. For example, allergen-free chicken meat and eggs can be developed by knocking out allergen-related genes such as ovalbumin and ovomucoid [113, 114]. In addition, it is possible to make double-muscled and muscle hypertrophy chickens by editing muscle-related genes such as myostatin, as is well-reported in other livestock [126–128]. Since conventional genetically modified organism (GMO) has foreign gene or uncontrolled random mutation, there has been public concern about the safety issue of food derived from GMO due to unknown allergen reaction or use of antibiotic resistance genes. On the other hand, genome-edited chickens and other livestock can be produced by controlled precise genome editing technology similar to mutations in intrinsic genomic sequences, like natural mutations, rather than foreign gene insertion as in conventional GMO. Thus, scientists and educators should convince the public that genome edited animals are similar to natural selected or conventional breeding programmed animal via natural mutation [129]. Through the public discussion and social consensus, genome edited animals are expected to be accepted by the consumers in the near future.

Additionally, birds are more likely to develop ovarian cancer than other animal model because they lay a large number of eggs for their lifecycle and have a relatively short ovulation cycle, therefore birds are considered to be one of the best animal model for studying human ovarian cancer [130]. Thus, with precise gene editing in ovarian cancer-related genes, it may be possible to create avian models similar to human ovarian cancer and to reveal the genetic mechanisms of ovarian cancer pathogenesis through gene editing technology. Although avian genome editing research has been conducted mostly in chickens, it will be possible to gradually apply it to various other birds in the near future. Most notably, zebra finches are an exclusive non-human model organism for investigating the biological basis of speech learning, and have been widely used for neurobehavioral research [131]. Zebra finches are also considered as the novel avian models for human diseases that cannot be easily studied in other animal models such as neurological behavior model, Huntington’s disease and vocal learning model [132–135]. Until recently, transgenic system in zebra finches usually utilize the virus-mediated system that directly injects viruses into the embryos [133]. Gene editing technology can be widely applied to reveal the function and mechanism of invaluable genes in zebra finches through the development of efficient germline transmission systems, including PGC-mediated or sperm-mediated delivery and other reliable strategies. In addition, we expect that it will be possible to control bird-specific diseases and develop avian disease-resistant birds through gene editing of pathogenesis-related genes in birds. In particular, high-risk infectious poultry diseases such as avian influenza and Marek’s disease cause serious problems in various countries and adversely affect the poultry industry. Although it will be necessary to first understand the disease mechanisms and host factors of avian viruses [136, 137], avian gene editing technology is expected to develop avian disease-resistant birds by eliminating host factors or receptors of avian viruses.

## Conclusion

Birds are not only important as a food resource, but also an ideal animal model for various disciplines such as behavioral science, immunology and developmental biology. Despite of their importance as an experimental model animal, until a few years ago, there were many challenges and difficulties in transgenesis and gene editing in birds. Recently developed programmable genome editing tools have facilitated a new era of avian models combined with PGC culture systems. It is expected to create innovative genome edited avian models, including specific-gene knockout avian models, allergen-free poultry, human disease model, egg-based bioreactor and avian disease resistance model. Although the establishment of germline-competent cell culture systems has not yet been successful in various birds, and challenges for developing efficient germline transmission strategies still remain, it will be possible to develop such a useful genome-edited avian models in the near future by efficiently introducing gene editing tools into the germline-competent cells of birds. Thus, application of gene editing technology to avian species will provide far more possibilities and benefits to humans.

## Abbreviations

bPGC: Embryonic blood-derived PGC
Cas9: CRISPR associated protein
cpf1: CRISPR from Prevotella and Francisella 1
CRISPR: Clustered regularly interspaced short palindromic repeat
crRNAs: Clustered regularly interspaced short palindromic repeat RNA
CVH: Chicken vasa homolog
DAZL: Deleted in azoospermia-like
EGK: Eyal-Giladi and Kochav
ESC: Embryonic stem cell
GFP: Green fluorescent protein
GMO: Genetically modified organisms
gPGC: Embryonic gonad-derived PGC
gRNA: CRISPR guide RNA
HDR: Homology directed repair
HH: Hamburger and Hamilton
JH: Joining gene segment of immunoglobulin heavy chain
NHEJ: Non-homologous end joining
PAM: Protospacer adjacent motif
PAS: Periodic acid schiff
PGC: Primordial germ cell
SSEA1: Stage specific embryonic antigen-1
TALEN: Transcription activator-like effector nuclease
tracrRNA: Trans-activating crRNA
VH: Variable gene segment of immunoglobulin heavy chain
ZFN: Zinc-finger nuclease
